# Habits of Cleanliness

**Published:** 1875-09

**Authors:** 


### HABITS OF CLEANLINESS.

A letter, warmly commendatory of a certain educational institution near Buffalo, known as "De Veaux College," is furnished for publication. In that letter we find the following:

"Everything at De Veaux is done on the stroke of the bell, which bell, by the by, is large and sonorous and warranted to wake the soundest sleeper. The first bell means 'get up and dress.' It rings again when the boys descend to the well-arranged lavatory where each has his place for washing, his brushes, comb, &c.; they then assemble for inspection roll call, after which they march to the breakfast-room. And here, as they stand before the tables to ask a blessing (which they sing) on the morning meal, one cannot but notice the blooming health of every individual boy. Boys who come here pale and weak and 'scrawny,' shambling in gait and with eyes as dull as putty, are soon transformed, by the magical influences of regular hours, healthful diet, out-door drill and exercise, the pure air of the locality and the habits of cleanliness soon acquired, if wanting."

And this system of marching up to a washbowl and applying a little water to the face and neck, is called a habit of cleanliness! When will teachers and parents and the Christian world comprehend that there is no real cleanliness which does not provide a daily private washing of the entire surface of the body. If you think you are cleanly when you only wash the hands and face—say less than five per cent. of the surface—consult the first heathen you meet and he will tell you of your error.

---

If a glass stopper won't move, hold the neck of the bottle to a flame, or warm it by taking two turns of a string and see-sawing it. The heat engendered expands the neck of the bottle before a corresponding expansion reaches the stopper.

Deaths from Bowel Diseases.—As we write these lines the report, no doubt, authentic, reaches us, that little children, chiefly infants, are dying in the city of New York, at the rate of one hundred per day. Every fifteen minutes a little one is taken off! The great destroyer of this season, is, of course, bowel-complaint. In 1873, excluding from the count the winter months, twenty-one thousand deaths occurred in this country from diarrhœa, dysentery and cholera infantum. We shall never know how many of these died from the disease, and how many from the effects of the opium given either by physicians or in some horrid "soothing" mixture. But this we do know; that for all bowel-complaints, in young children, opium in any form is sure death. We know of no medicine within the reach of everyone, which we can fully recommend in such cases, except the little sugar pellets, called the Van Deren Remedy. This will cure most cases, however severe, if followed up with good nursing.

---

It often happens that black calico, and other printed goods which have a white pattern on a black ground, will not bear washing unless some precautions are taken to prevent "running;" or, in other words, the white spots acquire a reddish color, and the black ground becomes dull and foxy. A case of this kind having occurred in Stuttgart, the dress was sent to the Royal Laboratory there, and the following method resorted to which proved successful. A sufficient quantity of water was put in a wash-boiler and heated to the boiling point. There were then dissolved in it fifty grams red chromate of potash, eighty grams common salt and sixty grams crystals of sal soda. The dress was put into this hot bath for five minutes and frequently turned and stirred, and then washed in clean water until the water spots appeared perfectly bright and clear.

